# Effect of sequential enteral nutrition support on elderly patients with severe ischemic stroke after thrombectomy

**DOI:** 10.3389/fmed.2025.1739299

**Published:** 2026-01-26

**Authors:** Lili Jiang, Qingmei Wang, Yang Zhang, Mengxia Ding, Zhenyuan Cai

**Affiliations:** 1 Department of Neurology II, The Affiliated Huai’an No. 1 People’s Hospital of Nanjing Medical University, Huai’an, Jiangsu, China; 2 Department of Nursing, The Affiliated Huai’an No. 1 People’s Hospital of Nanjing Medical University, Huai’an, Jiangsu, China

**Keywords:** elderly, immune function, ischemic stroke, sequential enteral nutrition support, thrombectomy

## Abstract

**Objective:**

To explore the effects of sequential enteral nutrition support in elderly patients with severe ischemic stroke after thrombectomy.

**Methods:**

From January 2022 to January 2024, 115 elderly patients with severe ischemic stroke who underwent thrombectomy were selected and divided into a control group (*n* = 57) and an intervention group (*n* = 58). The control group received routine enteral nutrition support, while the intervention group received sequential enteral nutrition support. The nutritional status, immune function, degree of neurological impairment, prognosis, daily living ability, gastrointestinal dysfunction, and incidence of complications were compared between the two groups.

**Results:**

Compared with the control group, the intervention group had higher levels of albumin (ALB), total protein (TP), prealbumin (PA), and hemoglobin (Hb) on the 14th day after intervention (*p* < 0.05), higher levels of immunoglobulin A (IgA), immunoglobulin M (IgM), and immunoglobulin G (IgG) on the 14th day after intervention (*p* < 0.05), lower NIHSS score after 14 days of intervention, higher Glasgow Coma Scale (GCS) score, higher Barthel Index (BI) at discharge (*p* < 0.01), lower gastrointestinal dysfunction score on the 14th day after intervention (*p* < 0.05), and lower complication rate (*p* < 0.05).

**Conclusion:**

Sequential enteral nutrition support can attenuate the deterioration of intestinal adaptability under pathological conditions, promote the absorption of nutrients, and slow the decline of nutritional status in elderly patients with severe ischemic stroke after thrombectomy in the short term. It also shows early functional benefits, such as mitigating the worsening of the GCS and NIHSS scores at 14 days and the BI at discharge, and reducing the occurrence of short-term complications. Additionally, it appears to decelerate the decline of cellular and humoral immune parameters. These short-term physiological and early functional modifications create favorable conditions for the initial treatment and early rehabilitation of the diseases.

## Introduction

Stroke is one of the main diseases causing disability and death in humans. Every 6 s, someone dies from a stroke, and every 6 s, someone becomes permanently disabled due to a stroke (1). Since 2015, stroke has become the leading cause of illness, death, and disability among Chinese residents, and is also the primary killer threatening people’s lives and health (2). Ischemic stroke accounts for approximately 80% of all strokes (3). The onset of ischemic stroke typically presents symptoms of neurological dysfunction such as sensory impairment, hemiplegia, aphasia, ataxia, and is often accompanied by brain symptoms such as coma, vomiting, and headache, posing a serious threat to life (4). At present, the most effective clinical treatment for ischemic stroke is to perform thrombectomy as soon as possible, which can unclog the blocked cerebral vessels of the patient, improve cerebral microcirculation, and be beneficial to the prognosis of the patient (5). However, in the early stage of thrombectomy, it is prone to cause reperfusion brain edema, extensive core infarction, and even cerebral hemorrhage, which can endanger life (6).

Clinical studies have shown that elderly patients with severe ischemic stroke who undergo thrombectomy often experience swallowing dysfunction or cognitive impairment, making it impossible for them to eat through the mouth, resulting in difficulties in nutrient intake. As a result, these patients are at risk of malnutrition (7). Enteral nutrition support is the most ideal nutritional support method for ischemic stroke patients with swallowing dysfunction or cognitive impairment (8). Compared with parenteral nutrition, enteral nutrition is more in line with the physiological characteristics of the patients (9). If enteral nutrition support is given to patients as early as possible, the nutritional status of the patients can be improved, and their rehabilitation can be promoted (10). However, elderly patients with severe ischemic stroke have different tolerance to enteral nutrition support due to neurological dysfunction, high metabolic state, insufficient nutrient intake, and low immunity (11). An excessively high concentration of enteral nutrition support may lead to intestinal-related complications (12). Sequential enteral nutrition support is the process of gradually transitioning from short peptide-type nutritional preparations to full protein-type nutritional preparations (13). Compared with traditional enteral nutrition support, sequential enteral nutrition support is a progressive process that enables patients to better adapt to enteral nutrition support, promotes the improvement of the patient’s nutritional level, and avoids the occurrence of related complications (14).

This study analyzed the impacts of sequential enteral nutrition support on elderly patients with severe ischemic stroke after thrombectomy, hoping to provide reference for clinical nursing of enteral nutrition support in elderly patients with severe ischemic stroke after thrombectomy.

## Data and methods

### Study design

This was a single-center randomized controlled study. One hundred and fifteen elderly patients with severe ischemic stroke after thrombectomy from January 2022 to January 2024 were divided into control group (*n* = 57) and intervention group (*n* = 58) following the random number table method. Inclusion criteria: (1) Patients met the indications for thrombectomy; (2) All patients were met the diagnostic criteria of ischemic stroke and was confirmed by CT and MRI; (3) Age ≥65 years old; (4) Glasgow Coma Scale (GCS) (15) ≤8 points; and (5) Patients or family members gave informed consent to this study and signed informed consent; Exclusion criteria: (1) Patients had abnormal cardiac, pulmonary and gastrointestinal dysfunction, coagulation dysfunction, and infection; (2) Patients with metabolic diseases; (3) Unable to cooperate with treatment and accompanying visitors for various reasons; and (4) Patients with gastrointestinal, liver, neurological and vascular diseases.

### Randomization and blinding

The random sequence was generated by an independent statistician who was not involved in the patient recruitment, treatment, or outcome assessment processes. A computer-based random number generator was used to create a list of random numbers. Each patient was assigned a unique number from this list, and based on whether the number was odd or even, they were allocated to either the control or the intervention group.

To ensure allocation concealment, we employed sealed, opaque envelopes. After the random sequence was generated, the assignments were placed inside these envelopes. The envelopes were sequentially numbered according to the order of patient enrollment. When a patient was ready to be randomized, an envelope was opened by a research assistant who was not involved in the clinical care of the patients or the outcome assessment. This way, the treating physicians and researchers were unaware of the group assignment until the envelope was opened, thus preventing any potential bias in patient selection or treatment.

Outcome assessors were blinded to the group assignments of the patients to minimize measurement bias. For the assessment of the National Institutes of Health Stroke Scale (NIHSS), GCS, Barthel Index (BI), and gastrointestinal dysfunction scale, as well as the recording of complications, the assessors were not informed whether a patient belonged to the control or intervention group. They conducted the evaluations based solely on the clinical presentation and test results of the patients, without having any knowledge of the treatment they had received. Additionally, the data analysts who performed the statistical analysis of the outcome data were also blinded to the group assignments to ensure the objectivity of the analysis.

### Sample size calculation

We conducted a sample size calculation prior to the start of the study. Based on previous similar studies and clinical experience, we used the mean BI score at discharge between the intervention group and the control group as the primary outcome (16). With a significance level of *α* = 0.05 (two-tailed), a power of 1−*β* = 0.80, and a 20% dropout rate, using the G*Power software, we calculated that the sample size for each group was 55 cases. In the actual study, we recruited a total of 115 patients, including 57 cases in the control group and 58 cases in the intervention group. This was sufficient to meet the sample size requirement for detecting the expected differences in the main indicators.

### Methods

After admission, patients in both groups received thrombectomy and routine nursing. Enteral nutrition support began to be given intranasal gastric tube or jejunal tube within 24 to 72 h of onset. All patients were provided with energy at 25 ~ 30 kcal/kg·d.

The control group was given routine enteral nutrition support. After admission, the patients were given a whole protein nutritional supplement (Nutricia, Netherland) at a rate of 20 mL/h gradually increased to 80 ~ 100 mL/h if no adverse reactions such as reflux, diarrhea, abdominal distension, and the total amount reached 1,000 ~ 1,500 mL/d. If there were no adverse reactions such as reflux, diarrhea, or abdominal distension, the rate was gradually increased to 80 to 100 milliliters per hour, until the total intake reached 1,000–1,500 mL/d.

The intervention group was given sequential enteral nutrition support. During the first 3 days, short peptide-type enteral nutrition (Nutricia, Netherland) and an enteral nutrition pump were used to control the speed, starting at 20 mL/h. If no adverse reactions such as reflux, diarrhea, and abdominal distension occurred, the dose was gradually increased to 80 ~ 100 mL/h until the total amount reached 1,000 ~ 1,500 mL/d. From the 4th day onwards, the whole protein type nutrient (Nutricia, Netherland) was used, with the injection method and dosage being the same as the short peptide-type enteral nutrition.

### Observation indicators

(1) Five mL fasting venous blood was collected from patients at the time of admission and 14 days after treatment. After centrifugation at 3000 r/min centrifugation for 10 min, the serum was separated. The levels of albumin (ALB), total protein (TP), prealbumin (PA) and hemoglobin (Hb) was detected using the Hitachi 7,600 automatic biochemical analyzer.
(2) Five mL fasting venous blood was collected from patients at the time of admission and 14 days after treatment. The levels of immunoglobulin (Ig) A, IgG and IgM were determined by enzyme-linked immunosorbent assay (ELISA).
(3) The NIHSS (17) and GCS scores were observed at the time of admission and 14 days after treatment. The NIHSS table assessed the degree of neurological impairment of patients from 11 aspects, including language, visual field, lower limb movement, facial palsy, dysarthria, ataxia, consciousness level, upper limb movement, sensation, neglect syndrome, and gaze, with a total score of 35. The higher the score, the more severe the functional impairment. The GCS assessed patients in three aspects of movement, eye opening, and language, with a total score of 15. The lower the score, the more severe the coma.
(4) Using BI (18), the patient’s daily living ability was evaluated at the time of discharge. The full score was 100, and the higher the score, the higher the self-care ability.
(5) Gastrointestinal dysfunction scale was used to evaluate the gastrointestinal function of the two groups. This scale included 5 indicators, including clinical manifestations, bowel sounds, mucosal lesions, intestinal absorption area, and bacterial displacement. Each indicator was scored on a 4-point scale from 1 to 4, with a maximum score of 20. The higher the score, the more severe the gastrointestinal dysfunction. The scale was developed and validated in previous research (19). The validation process involved a series of rigorous steps, including initial item generation based on clinical expertise and literature review, followed by pilot testing to assess the reliability and validity of the scale in a relevant patient population. Further large-scale studies were conducted to confirm its ability to accurately measure gastrointestinal dysfunction across different settings and patient groups. There are no pre-defined strict cut-off values for this scale in our study. Instead, the total score is used as a continuous variable to represent the degree of gastrointestinal dysfunction, with higher scores indicating greater severity. However, in clinical practice, based on previous experience and expert consensus, a score of 15–20 can be considered as indicating severe gastrointestinal dysfunction, 10–14 as moderate, and 1–9 as mild. Bowel sounds were assessed by trained medical staff (nurses or doctors) using a stethoscope. The assessment was carried out in a quiet environment, and the frequency, intensity, and character of the bowel sounds (such as normal, hyperactive, hypoactive, or absent) were recorded. Based on these characteristics, a score was assigned according to predefined criteria within the scale. The detection of bacterial translocation was mainly based on laboratory tests. Blood, mesenteric lymph nodes, or other relevant tissue samples were collected under sterile conditions. These samples were then cultured and analyzed for the presence of bacteria that are normally resident in the gut but have translocated to other sites. If bacterial translocation was detected, a higher score was assigned according to the scale; if not, a lower score was given. This assessment was typically performed by laboratory technicians following standard microbiological procedures. Mucosal lesions were evaluated through endoscopic examination (such as gastroscopy or colonoscopy, depending on the suspected site of the lesion). Trained endoscopists conducted the examinations and visually inspected the mucosal surface for any signs of damage, such as erosions, ulcers, or bleeding. The severity and extent of the mucosal lesions were then graded according to the scale’s criteria, and a corresponding score was assigned.
(6) During the intervention period, the incidence of complications in both groups was observed, including gastrointestinal bleeding, diarrhea, infection, and reflux aspiration. The following clear diagnostic criteria for each type of complication was as follows: Gastrointestinal bleeding: Vomiting blood (bright red or coffee-ground-like blood), black stool (indicating upper gastrointestinal bleeding, black, pasty stool), or fresh blood passing through the rectum (usually related to lower gastrointestinal bleeding). Diarrhea: Passage of three or more loose or liquid stools per day, with a consistency significantly different from the patient’s normal bowel movements (e.g., watery or very soft). The duration is at least two consecutive days. Infection: Fever (body temperature > 38.0 °C), chills, fatigue, and an increased white blood cell count (> 10 × 10^9^/L). Reflux aspiration: Sudden coughing, choking or wheezing during or after feeding, usually accompanied by a history of gastric content reflux. Chest X-ray examination may show pulmonary infiltrates, while bronchoscopy can clearly see foreign bodies in the airways.

### Statistical analysis

SPSS 20.0 software was adopted for statistical processing of relevant data. We first checked the normality of the data using the Shapiro–Wilk test. The results were expressed as mean ± standard deviation (x ± s). If the data followed a normal distribution and the variances between groups were homogeneous, we used the independent-samples *t*-test to compare the differences between the control group and the intervention group. If the data did not follow a normal distribution, we used the non-parametric Mann–Whitney U test for between-group comparisons. To assess the changes within each group over time, we employed repeated measures analysis of variance (ANOVA) to compare the differences in each variable at different time points as well as the differences between the two groups. Effect sizes were calculated by 95% confidence intervals (95% CI). Statistical data expressed as frequency and percentage were compared using Fisher’s Exact Test. A *p*-value less than 0.05 was considered to indicate that the difference was statistically significant.

## Results

### General data of patients in 2 groups

It was manifested in Table 1 that, the general data of patients had no difference between 2 groups (*p* > 0.05), implying comparability.

**Table 1:** General data of patients in 2 groups.

Groups	Cases	Gender		Age (years)	GCS score (points)
		Male	Female		
Control group	57	30 (52.63)	27 (47.37)	65.12 ± 8.24	6.21 ± 0.75
Intervention group	58	30 (51.72)	28 (48.28)	65.16 ± 8.17	6.18 ± 0.73
*P*		>0.999		0.979	0.828

### Nutritional status between 2 groups

**Figure 1:**
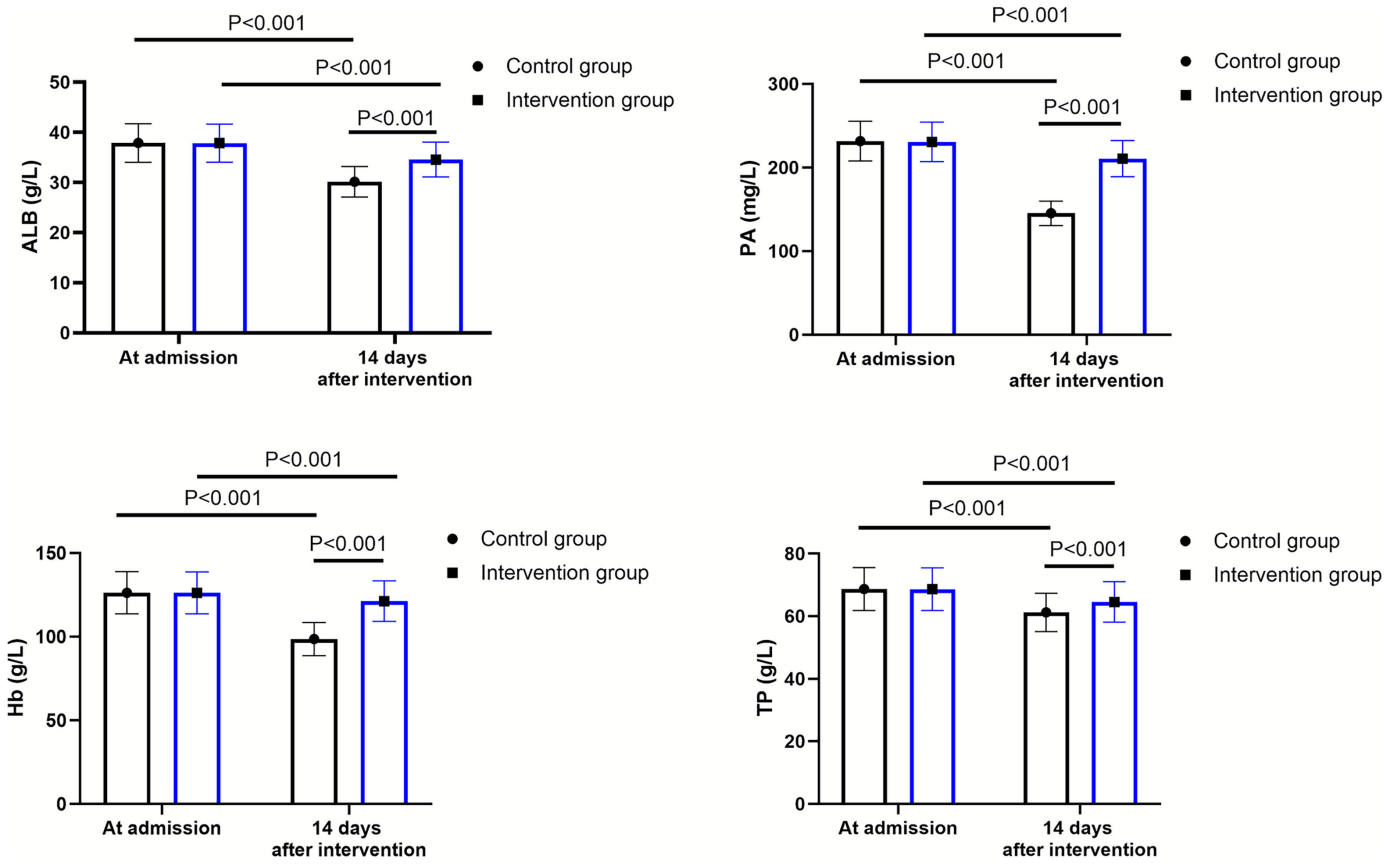
Nutritional status between the control group (*n* = 57) and the intervention group (*n* = 58). Data were expressed as mean ± standard deviation (*x* ± *s*).

As shown in Figure 1, at admission, there were no significant differences in the levels of ALB, PA, Hb and TP between the two groups (*p* > 0.05). Fourteen days after the intervention, the levels of ALB, PA, Hb and TP declined in both groups (*p* < 0.001, 95% CI: −3.119–1.271; *p* < 0.001, 95% CI: −37.72–26.67; *p* < 0.001, 95% CI: −14.39–8.215; *p* < 0.001, 95% CI: −3.373–0.053). Compared to the control group, the intervention group had higher levels of ALB (*Δ* difference = 4.43 g/L), PA (Δ difference = 65.42 mg/L), Hb (Δ difference = 22.67 g/L) and TP (Δ difference = 3.35 g/L) 14 days after intervention (*p* < 0.001, 95% CI: 4.581–6.429; *p* < 0.001, 95% CI: 47.71–58.76; *p* < 0.001, 95% CI: 13.21–19.39; *p* < 0.001, 95% CI: 4.047–7.473).

### Immune function between 2 groups

**Figure 2:**
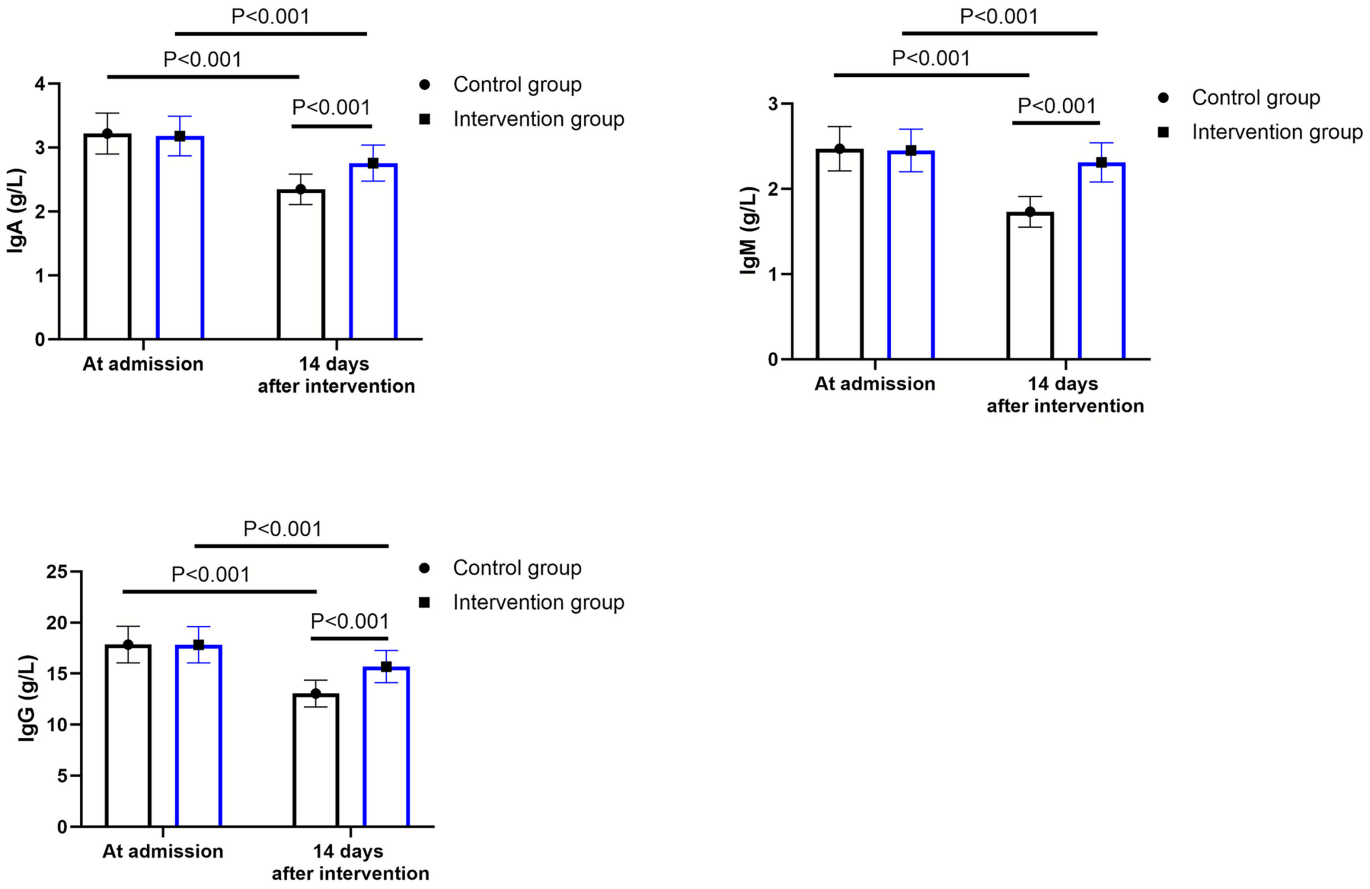
Immune function between the control group (*n* = 57) and the intervention group (*n* = 58). Data were expressed as mean ± standard deviation (*x* ± *s*).

As shown in Figure 2, at admission, there were no significant differences in the levels of IgA, IgM and IgG between the two groups (*p* > 0.05). Fourteen days after intervention, the levels of IgA, IgM and IgG declined in both groups (*p* < 0.001, 95% CI: −0.260–0.109; *p* < 0.001, 95% CI: −0.340–0.219; *p* < 0.001, 95% CI: −1.727–0.882). Compared to the control group, the intervention group had higher levels of IgA (*Δ* difference = 0.41 g/L), IgM (Δ difference = 0.58 g/L) and IgG (Δ difference = 2.63 g/L) 14 days after intervention (*p* < 0.001, 95% CI: 0.569–0.720; *p* < 0.001, 95% CI: 0.379–0.500; *p* < 0.001, 95% CI: 3.053–3.897).

### NIHSS and GCS scores between 2 groups

**Figure 3:**
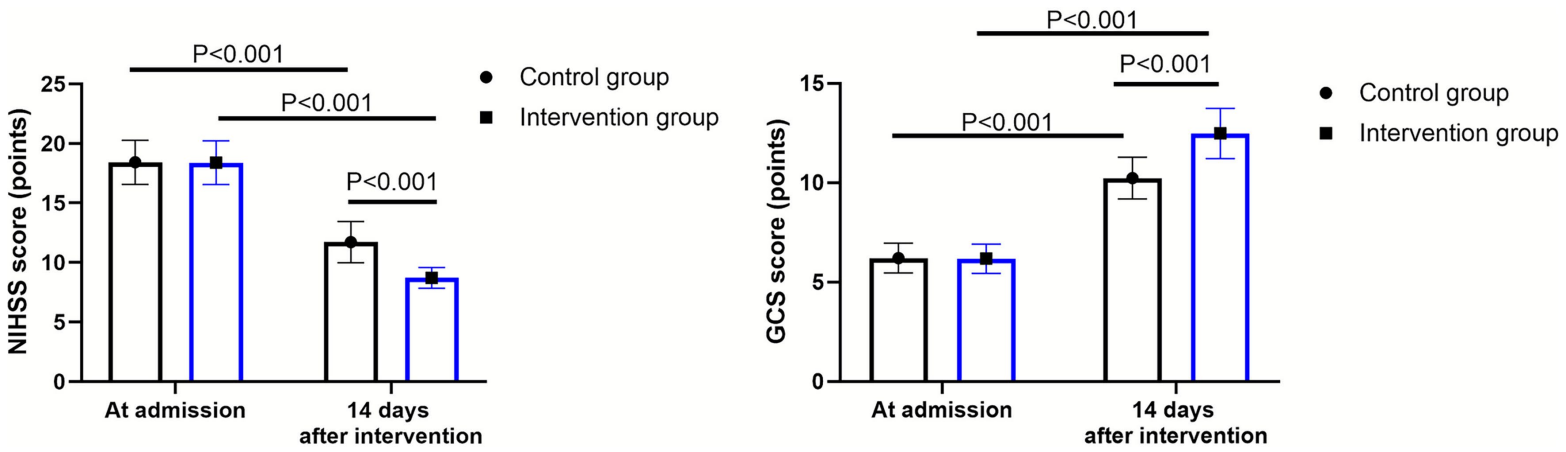
NIHSS and GCS scores between the control group (*n* = 57) and the intervention group (*n* = 58). Data were expressed as mean ± standard deviation (*x* ± *s*).

As shown in Figure 3, at admission, there were no significant differences in NIHSS and GCS scores between the two groups (*p* > 0.05). Fourteen days after intervention, the NIHSS score declined while the GCS score elevated in 2 groups (*p* < 0.001, 95% CI: 1.099–1.941; *p* < 0.001, 95% CI: −1.363–0.857). Compared to the control group, the intervention group had lower NIHSS score (*Δ* difference = 10.84 points) and higher GCS score (Δ difference = 2.25 points) 14 days after intervention (*p* < 0.001, 95% CI: 7.759–8.601; *p* < 0.001, 95% CI: −5.413–4.907).

### Patient’s daily living ability between 2 groups

**Figure 4:**
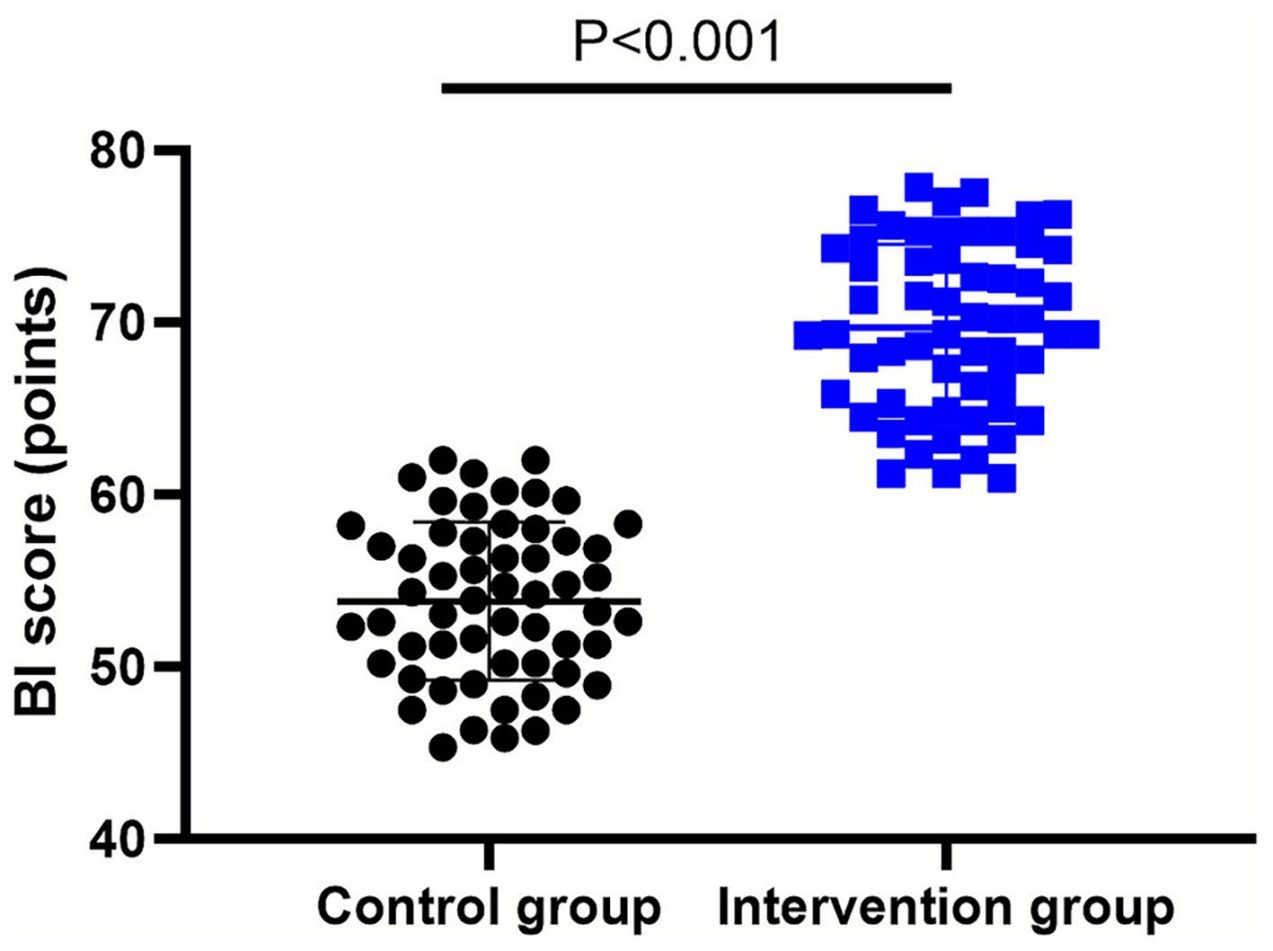
Patient’s daily living ability between the control group (*n* = 57) and the intervention group (*n* = 58). Data were expressed as mean ± standard deviation (*x* ± *s*).

It was manifested in Figure 4 that, relative to the control group, the intervention group presented higher BI score at discharge (*p* < 0.001, 95% CI: 14.15–17.62).

### Gastrointestinal dysfunction between 2 groups

**Figure 5:**
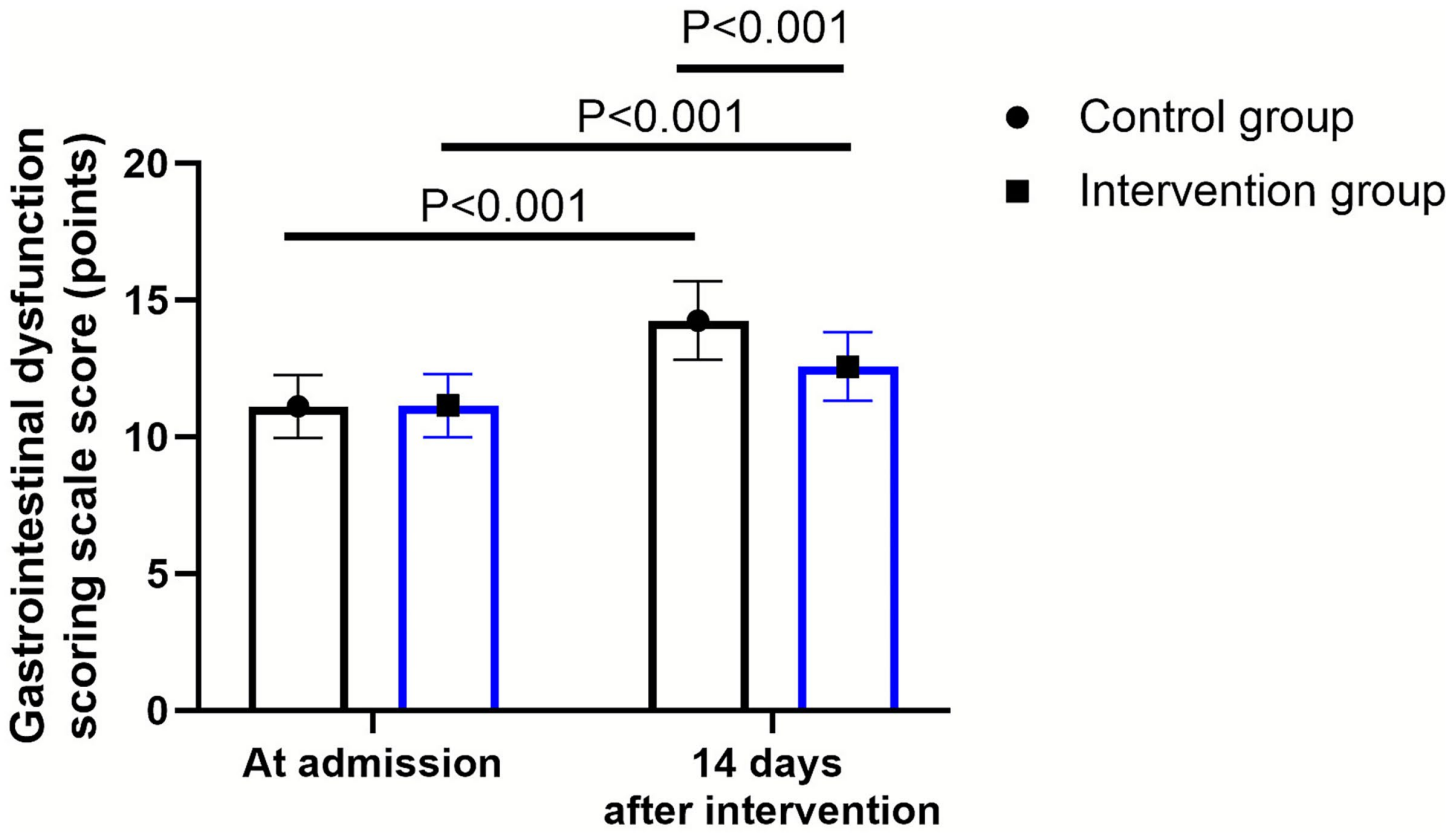
Gastrointestinal dysfunction between the control group (*n* = 57) and the intervention group (*n* = 58). Data were expressed as mean ± standard deviation (*x* ± *s*).

As shown in Figure 5, at admission, there were no significant differences in gastrointestinal dysfunction scale score between the two groups (*p* > 0.05). Fourteen days after intervention, the gastrointestinal dysfunction scale scores elevated in 2 groups (*p* < 0.001, 95% CI: 0.498–1.151). Compared to the control group, the intervention group had lower gastrointestinal dysfunction scale scores 14 days after intervention (*Δ* difference = 1.68 points, *p* < 0.001, 95% CI: −2.611–1.959).

### Incidence of complications between 2 groups

It was manifested in Table 2 that, relative to the control group, the intervention group had lower incidence of complications (*p* < 0.05).

**Table 2:** Incidence of complications between 2 groups.

Groups	Cases	Gastrointestinal bleeding	Diarrhea	Infection	Reflux aspiration	Total incidence rate
Control group	57	2 (3.51)	4 (7.02)	3 (5.26)	3 (5.26)	12 (21.05)
Intervention group	58	1 (1.72)	1 (1.72)	1 (1.72)	1 (1.72)	4 (6.88)
*P*						0.033

## Discussion

Globally, stroke is the second leading cause of death after ischemic heart disease (20). In China, stroke is the leading cause of death, with ischemic stroke accounting for over 75% of all strokes cases, and having become a serious social and public health issue (21). Recently, with the continuous development of medical technology, early diagnosis and treatment of ischemic stroke have been effectively advanced. Among them, thrombectomy is widely used due to its advantages such as long treatment window and high vascular opening rate, which has to some extent reduced the risk of death for patients and improved their prognosis (22).

The elderly with severe ischemic stroke are in a coma state and unable to actively eat. They have difficulty swallowing, insufficient protein and calorie intake, and the stress response consumes a large amount of energy and protein, increasing catabolism and causing negative nitrogen balance (23). The lack of essential amino acids, fatty acids, trace elements and other nutrients in the body can lead to low immunity, prone to infections, decreased immune function, and the occurrence of various complications, thereby increasing the mortality and disability rates of patients and increasing their burden (24). Studies have shown that the incidence of postoperative malnutrition in patients with ischemic stroke is approximately 15%, and it can rise to 30% 1 week later (25). Nutritional support is considered to be an important part of treating severe ischemic stroke, which helps to improve metabolic disorders caused by insufficient nutrition and the ischemic hypoxia of brain tissue, reduce secondary damage, reduce complications, lower mortality, accelerate the recovery of neurological function, and improve prognosis (26).

Patients with severe ischemic stroke usually do not have obvious organic lesions in the gastrointestinal tract and meet the conditions for early implementation of enteral nutrition support (27). Studies have shown that enteral nutrition is more consistent with the physiological state of the human body than intravenous nutrition, and can significantly reduce complications caused by parenteral nutrition, such as infection and internal environment disorders (28). Due to the highly stressful state of patients with severe ischemic stroke, there are conditions such as contraction of internal organs’ blood vessels, ischemia and hypoxia of the gastrointestinal mucosa, mucosal edema, damage to the cytoskeleton of mucosal epithelial cells, and damage to the intestinal barrier, which lead to bacterial translocation, increased infection rate, obstruction of neurological function recovery, and increased mortality (29). How to provide appropriate feeding is a problem that neurologists need to solve.

In this study, sequential enteral nutrition support was adopted to provide nutritional support for elderly patients with severe ischemic stroke after thrombectomy. Sequential enteral nutrition support refers to the first administrating short peptide-based enteral nutrition preparations, and then gradually transitioning to full protein-based enteral nutrition (30). Considering the metabolic characteristics of elderly patients with severe ischemic stroke, in this study, short peptide-based enteral nutrition was given for the first 3 days, followed by gradually providing full protein-based enteral nutrition, and the sequential enteral nutrition support mode was gradually increased from a low dose to a full dose.

Our research results indicate that, compared with the control group, the intervention group had higher levels of ALB, TP, PA and Hb 14 days after the intervention. This suggests that sequential enteral nutrition support can alleviate the gradual increase in protein and calorie consumption of patients, reduce the degree of malnutrition, and promote the absorption of nutrients. At the same time, it can slow down the decline in the nutritional status of elderly patients with severe ischemic stroke. This was consistent with previous reports (31, 32). At the same time, the nutritional indicators of the two groups of patients decreased compared to those on the first day at the 14th day. This is because that in the early stage of severe ischemic stroke, gastrointestinal peristalsis is weakened, intestinal absorption function is reduced, and factors such as stress ulcers are affected. Moreover, it is difficult to administer full enteral nutrition solutions in the early stage (33). During the process of implementing enteral nutrition support, we found that patients could not reach full enteral nutrition support within 1 week after thrombectomy. This process may be prolonged over time. Patients may gradually recover to their pre-illness nutritional status, or they may achieve the predetermined feeding volume through indwelling naso-intestinal tube and jejunum nutrition tubes in the early stage.

After a severe ischemic stroke occurs, the body rapidly transitions into an immunosuppressive state. This is primarily characterized by a marked reduction in both the quantity and function of helper T lymphocytes, a decline in interleukin 2 secretion, a diminished capacity to augment the killing effect of CTL cells, and impaired humoral immune function (34). In our study, the results indicated that compared with the control group, the intervention group had higher levels of IgA, IgM, and IgG within 14 days after the intervention. However, it should be noted that both groups experienced a decrease in immunoglobulin levels over the 14-day period, with the intervention group showing a relatively smaller decline. This suggests that sequential enteral nutrition support was associated with a less pronounced reduction in serum immunoglobulins. We initially hypothesized that sequential enteral nutrition support might significantly enhance overall immune function, encompassing both cellular and humoral immunity. The potential mechanism could involve the improvement of immune function through the regulation of the intestinal barrier and the enhancement of intestinal secretory IgA (35).

Besides, our study also discovered that compared with the control group, the intervention group had a lower NIHSS score and a higher GCS score 14 days after intervention, and a higher BI score at discharge. As the nutritional status improved or was maintained, the patient’s metabolism was in a relatively stable state, and both nutrition and stress conditions were improved, resulting in alleviation of brain injury and better recovery after discharge (36).

In addition, our study revealed that compared with the control group, the intervention group had a lower score for gastrointestinal dysfunction 14 days after the intervention, and a lower incidence of complications. This indicates that sequential enteral nutrition support can improve the gastrointestinal tolerance of elderly patients and reduce the occurrence of gastrointestinal dysfunction and complications. The main reason is that continuous enteral nutrition support can enhance the patient’s gastrointestinal tolerance and reduce the number of interruptions in enteral nutrition through gradual adjustment of dosage and speed (37). For elderly patients with ischemic stroke, their gastrointestinal function will decline. The nutritional supply not only needs to maintain calories and positive nitrogen balance, but also needs to maintain cellular metabolism and improve organ function. It is necessary to maintain the nutritional supply and ensure the comfort of the nutritional supply, while avoiding gastrointestinal discomfort symptoms such as gastric retention, reflux, and vomiting during the feeding process (38).

However, several limitations of our study should be explicitly acknowledged, which necessitate more cautious interpretation of the findings and frame them as hypothesis-generating rather than definitive. First, our follow-up period was relatively short. We only measured biomarkers at 14 days and the Barthel Index at discharge, without assessing any long-term outcomes. This limited follow-up restricts our ability to evaluate the long-term effects of sequential enteral nutrition support on patients’ recovery, such as its impact on long-term functional independence, quality of life, and the risk of recurrent stroke. Second, the baseline data collected in our study were somewhat limited. We did not adjust for some important confounders that could potentially influence the study results, such as patients’ pre-existing comorbidities, medications, and socioeconomic status. These unadjusted confounders may introduce bias and affect the accuracy of our conclusions regarding the effectiveness of sequential enteral nutrition support. Third, there was an incomplete description of the enteral formulas used in the study. The lack of detailed information about the enteral formulas makes it difficult for other researchers to replicate our study and compare the results with other similar studies. Fourth, selection bias may have been introduced during the patient recruitment process, as patients who were more likely to benefit from the intervention or had better baseline characteristics may have been preferentially enrolled. In addition, our study was not registered in a public trial registry. Trial registration is an important step in ensuring the transparency and integrity of clinical research. The lack of registration may raise concerns about the potential for selective reporting of results and other ethical issues. In light of these limitations, further well-designed, large-scale, long-term, randomized controlled trials with comprehensive baseline data collection, detailed descriptions of interventions, and trial registration are needed to confirm and expand upon our findings.

In conclusion, our exploratory study demonstrates that sequential enteral nutrition support can attenuate the deterioration of intestinal adaptability under pathological conditions, promote the absorption of nutrients, and slow the decline of nutritional status in elderly patients with severe ischemic stroke after thrombectomy in the short term. It also shows early functional benefits, such as mitigating the worsening of the GCS and NIHSS scores at 14 days and the BI at discharge, and reducing the occurrence of short-term complications. Additionally, it appears to decelerate the decline of cellular and humoral immune parameters. These short-term physiological and early functional modifications create favorable conditions for the initial treatment and early rehabilitation of the diseases.

## Data Availability

The datasets presented in this study can be found in online repositories. The names of the repository/repositories and accession number(s) can be found in the article/supplementary material.
